# Conservation of centromeric histone 3 interaction partners in plants

**DOI:** 10.1093/jxb/eraa214

**Published:** 2020-05-05

**Authors:** Burcu Nur Keçeli, Chunlian Jin, Daniel Van Damme, Danny Geelen

**Affiliations:** 1 Ghent University, Department Plants and Crops, unit HortiCell, Coupure Links, Ghent, Belgium; 2 Ghent University, Department of Plant Biotechnology and Bioinformatics, Technologiepark, Ghent, Belgium; 3 VIB Center for Plant Systems Biology, Technologiepark, Ghent, Belgium; 4 Cardiff University, UK

**Keywords:** CENH3, centromere, chromosome, haploid induction, post-translational modification, protein interaction

## Abstract

The loading and maintenance of centromeric histone 3 (CENH3) at the centromere are critical processes ensuring appropriate kinetochore establishment and equivalent segregation of the homologous chromosomes during cell division. CENH3 loss of function is lethal, whereas mutations in the histone fold domain are tolerated and lead to chromosome instability and chromosome elimination in embryos derived from crosses with wild-type pollen. A wide range of proteins in yeast and animals have been reported to interact with CENH3. The histone fold domain-interacting proteins are potentially alternative targets for the engineering of haploid inducer lines, which may be important when CENH3 mutations are not well supported by a given crop. Here, we provide an overview of the corresponding plant orthologs or functional homologs of CENH3-interacting proteins. We also list putative CENH3 post-translational modifications that are also candidate targets for modulating chromosome stability and inheritance.

## Teaser

Here we have listed 36 human and yeast CENH3 interaction partners and their corn, rice, and Arabidopsis homologs that are potentially interacting with plant CENH3.

## Introduction: CENH3 as a core component of centromeres

The histone H3 variant centromeric histone 3 (CENH3) is a component of the centromeric nucleosomes in eukaryotes (McKinley and Cheeseman, 2016). The role of CENH3 in nucleosome formation is conserved in yeast, mammals, and plants, but, compared with other histones, its amino acid sequence is poorly conserved (Drinnenberg *et al.*, 2016) and specific names were given: CENTROMERE PROTEIN A (CENPA) in mammals, CENTROMERE PROTEIN 1 (CNP1) in *Schizosaccharomyces pombe* and CHROMOSOME SEGREGATION 4 (CSE4) in *Saccharomyces cerevisiae* (Shrestha *et al.*, 2017). In *Arabidopsis thaliana* it was previously named HRT12 (Talbert *et al.*, 2002), but in more recent papers it is now named CENH3. For clarity, we use in this review the common name CENH3 to discuss general properties and extend it with the specific name in superscript when addressing species-specific features.

CENH3 loading onto the centromeres is of key importance for the ensuing establishment of the kinetochore (McKinely and Cheeseman, 2016; Sandmann *et al.*, 2017) and ensuring the fidelity of chromosome segregation during mitosis (Shrestha *et al.*, 2017). Specialized histone chaperones selectively bind centromeric histone and mediate the assembly of the centromeric nucleosomes (Zasadzińska and Foltz, 2017). The loading of CENH3^CENPA^ onto centromeres takes place during the G_1_ phase of the cell cycle when it complexes with histone H4 and nucleophosmin, and assembles the centromeric nucleosomes with the help of the chaperone HOLLIDAY JUNCTION RECOGNITION PROTEIN (HJURP) (Dunleavy *et al.*, 2009; Foltz *et al.*, 2009). CENH3^CENPA^ nucleosome assembly depends on a protein complex consisting of Mis18α, Mis18β, and KINETOCHORE NULL 2 (KNL2^M18BP1^), recruiting HJURP to the centromeres (Dunleavy *et al.*, 2009; Foltz *et al.*, 2009). The Mis18–KNL2^M18BP1^ complex does not, however, directly interact with CENH3^CENPA^ (Hayashi *et al.*, 2004; Fujita *et al.*, 2007). While KNL2^M18BP1^ mediates the recruitment of Mis18 proteins to the centromere (Fujita *et al.*, 2007), Mis18 proteins restrict the deposition of CENH3^CENPA^ to the centromeres (Nardi *et al.*, 2016).

The histone fold domain (HFD) of CENH3^CENPA^ contains a centromere-targeting domain (CATD) that is responsible for binding HJURP (Foltz *et al.*, 2009). In yeast, HJURP^SCM3^ and the CENH3^CNP1^ histone chaperone NASP^SIM3^ are involved in centromeric nucleosome assembly (Dunleavy *et al.*, 2007; Pidoux *et al.*, 2009). An ortholog of NASP identified in *A. thaliana* shows H3 chaperone activity (Maksimov *et al.*, 2016). NASP also binds CENH3, and NASP down-regulation impairs the loading of CENH3 at the centromeres (Le Goff *et al.*, 2020). An HJURP-like CENH3-selective chaperone has hitherto not been identified in plants.

CENH3 is assembled into nucleosome complexes with histone 2A, histone 2B, and histone 4, substituting the canonical histone H3 complex (Ramachandran and Henikoff, 2016). As in most eukaryotes, the plant centromeres are defined by the occurrence of arrays of CENH3 nucleosomes mixed with arrays of H3 nucleosomes (Panchenko *et al.*, 2011). Most of the centromeric histone-interacting proteins described in yeast and animals have not been identified in plants (Drinnenberg *et al.*, 2016), and for many candidate CENH3-interacting proteins experimental evidence for their role in CENH3 loading is lacking (Lermontova *et al.*, 2015). In addition to chaperones and other CENH3-interacting proteins orchestrating its deposition, there is mounting evidence for RNA transcribed from centromeric repeat sequences in specifying the centromeric chromatin (Talbert and Henikoff, 2018). Transcripts originating from the centromeric region are associated with the loading of centromeric nucleosomes and the stabilization of kinetochore proteins (Talbert and Henikoff, 2018). As neither the centromere sequence nor the CENH3 amino acid sequences are strictly conserved (Drinnenberg *et al.*, 2016) and even divergent CENH3s are interchangeable between some plant species (Maheshwari *et al.*, 2017), epigenetic factors including DNA methylation and chromatin modification are put forward as the determining regulators of CENH3 loading and maintenance.

The fidelity of chromosome segregation is impaired in animals and yeast cells by mutations that affect CENH3 loading and stability (Chen *et al.*, 2000; Pidoux *et al.*, 2003; Tanaka *et al.*, 2009; Ranjitkar *et al.*, 2010; Au *et al.*, 2013; Shrestha *et al.*, 2017). Loading of CENH3 to the centromeric DNA mainly depends on the C-terminally positioned HFD of CENH3 rather than its variable N-terminal tail (Sullivan *et al.*, 1994). However, a higher incidence of chromosome mis-segregation has been shown in yeast carrying mutations in the N-terminal tail of CENH3^CSE4^ (Chen *et al.*, 2000) that is not directly associated with the loading of CENH3 to the centromeres (Ravi *et al.*, 2010). Conversely, more stable association of the CENH3^CSE4^ with the centromeres via reduced ubiquitination at the N-terminal tail also leads to defects in chromosome segregation (Au *et al.*, 2013). Loading of the appropriate amount of CENH3 (Régnier *et al.*, 2005; Au *et al.*, 2008; Shrestha *et al.*, 2017) and/or tight regulation of the dynamics of CENH3 centromere interaction (Ohzeki *et al.*, 2016; Bui *et al.*, 2017) are therefore critical for ensuring kinetochore function and faithful segregation of the chromosomes.

Strict regulation of CENH3 loading on centromeres also plays a vital role in chromosome segregation in plants. The mitotic division rate is reduced in CENH3-targeting RNAi lines, whereas chromosome segregation defects were recorded in meiotic cells (Lermontova *et al.*, 2011). More recent findings from maize demonstrate the vital importance of strict regulation of CENH3 abundance. Overexpression of CENH3 results in lethality in maize callus, whereas green fluorescent protein (GFP)–CENH3 or CENH3–yellow fluorescent protein (YFP) overexpression is tolerated (Feng *et al.*, 2019). Both GFP–CENH3 and CENH3–YFP overexpression lines exhibit reduced deposition of the fusion proteins to maize centromeres (Feng *et al.*, 2019). C-terminal GFP or YFP fusions of CENH3 do not fully function in maize and *A. thaliana* somatic cells (De Storme *et al.*, 2016; Feng *et al.*, 2019), and several mutations in HFD reportedly cause chromosome elimination (Karimi-Ashtiyani *et al.*, 2015; Kuppu *et al.*, 2015). N-terminal tail modifications on the other hand result in chromosome elimination in plants (Ravi and Chan, 2010; Kelliher *et al.*, 2016).

## Haploid induction through impaired CENH3 functioning

Selection and fixation of desired traits is central to crop breeding. To breed a wide collection of vigorously growing hybrids, doubled haploids are created carrying two identical genome copies of the haploid parent (Maluszynski, 2003). These doubled haploids are crossed to generate new, potential elite, hybrids. In *A. thaliana*, the expression of a CENH3 variant with the GFP-tagged N-terminal tail of histone 3.3 (H3.3) fused to the HFD of CENH3, referred to as ‘tailswap’, expressed in the CENH3 knock-out mutant *cenh3-1*, produces 25–45% haploids upon crossing with the wild type (Ravi and Chan, 2010). The expression of N-terminal GFP–CENH3 fusion protein in the *cenh3-1* mutant background also results in 5% maternal haploid induction capacity (Ravi and Chan, 2010). Thus one might conclude that the N-terminal tail of CENH3 has an important role in haploid induction. Specific mutations in the C-terminal HFD of CENH3, however, also evoke chromosome elimination. Depending on the mutation, the efficacy was ~1–2% and ~12%, conferring on the HFD domain some importance (Karimi-Ashtiyani *et al.*, 2015; Kuppu *et al.*, 2015). The expression of a similar CENH3-tailswap construct in maize was shown to induce the formation of haploid progeny and suggests that it is a conserved mechanism that can be applied in other crops (Kelliher *et al.*, 2016). CenH3 mutation- and modification-based haploid induction strategies in plants are reviewed in more detail in Britt and Kuppu (2016), Wang and Dawe (2018), and Wang *et al.* (2019).

## CENH3 in species hybridization

The high accessibility of many flower structures allows for cross-pollination and requires the plant sexual reproduction system to establish multiple layers of hybridization barriers, one of which is interchromosome incompatibility mediated by the CENH3–centromere interaction (Tan *et al.*, 2015). Barley doubled haploids have been produced with a strategy called the ‘Bulbosum method’ based on interspecific crosses of *Hordeum vulgare* (cultivated barley) with *Hordeum bulbosum* (bulbous barley grass) (Houben *et al.*, 2011). In support of a role for CENH3 in rescinding hybridization events, interspecific crosses of *H. vulgare*×*H. bulbosum* result in paternal chromosome elimination during early embryogenesis following the loss of CENH3 from the centromeres of the paternal chromosomes (Sanei *et al.*, 2011). The capacity to eliminate foreign chromosomes is transferable as expression of a CENH3 orthologous sequence derived from a different species such as maize in *A. thaliana* shows chromosome elimination when crossed with pollen carrying the original CENH3 locus (Maheshwari *et al.*, 2015). This inability to transmit chromosomes loaded with ectopic CENH3 upon crosses with the wild type indicates that the native CENH3–centromere interaction harbors species-specific characteristics. Thus the elimination of chromosomes is based on the incongruence of the different centromere–CENH3 interactions.

## Conserved putative CENH3 interaction partners

Several candidate proteins interacting with the centromere have been reported, which are potentially involved in controlling the CENH3–centromere specificity. One of the well-studied examples is KNL2. KNL2 is required for CENH3^CENPA^ incorporation into chromatin, and CENH3^CENPA^ and KNL2 coordinately regulate chromosome condensation, kinetochore assembly, and chromosome segregation (Maddox *et al.*, 2007). A homolog of KNL2 has been identified in *A. thaliana* (Lermontova *et al.*, 2013). KNL2 knock-out mutants display varying defects in organ development and leaf shape, and show reduced fertility. These defects are attributed to alterations in chromosome structure and dynamics during cell division (Lermontova *et al.*, 2013). KNL2 contains a CENPC conserved motif (CENPC-k) that is required for centromeric localization (Sandmann *et al.*, 2017), and specific mutations in the CENPC-k motif lead to the production of haploid progeny upon crossing with wild-type pollen. These properties indicate that KNL2 is critical in establishing the CENH3–centromere interaction. In line with its role in controlling CENH3 abundance at the centromere, mutations in the CENPC-k motif of KNL2 lead to the production of haploid progeny (Lermontova, 2019).

By screening the literature reporting CENH3^CENPA/CNP1/CSE4^ candidate interacting proteins described for human CENH3^CENPA^, fission yeast CENH3^CNP1^, and budding yeast CENH3^CSE4^, we generated a list of 36 putative orthologs or functional homologs in *A. thaliana*, *Zea mays*, and *Oryza sativa* (Table 1). Histones were excluded from the selection because they are as such components of the centromeric nucleosomes. Affinity purification experiments, immunopurification coupled with western blot or MS, yeast-two hybrid, fluorescence resonance energy transfer (FRET), conditional growth arrest experiments, and data showing that misexpression changes the abundance of CENH3^CENPA/CNP1/CSE4^ at the centromeres were all considered as indications for interactions with CENH3, either direct or indirect, for example as a part of a protein complex. Candidate plant homologs were identified using reciprocal BLAST searches and the ‘HomoloGene’ database of NCBI (shown in bold in Table 1). Candidate plant sequences were either previously reported as functional analogs (underlined in Table 1) or no records were found (no markup, Table 1).

**Table 1.** Putative conserved interaction partners of CENH3 in *A. thaliana*, *O. sativa*, and *Z. mays*

*S. pombe*	*A. thaliana*	*O. sativa*	*Z. mays*	References
Ams2	Gata5:At5g66320^*a*^	Gata6:Os04g0539500	Gata3:Zm00001d017409	Chen *et al.* (2003); Takayama *et al.* (2016)
	Gata6:At3g51080		Gata6:Zm00001d025953	
	Gata7:At4g36240			
Hos2	Hda9:At3g44680	Hda9:Os04g0409600	Hda102:Zm00001d003813	Kobayashi *et al.* (2007)
Mis16	**Msi1:At5g58230** ^*b*^	Msi1:Os03g0640100	Msi1:Zm00001d033248	Hayashi *et al.* (2004)
Pob3	**Ssrp1:At3g28730**	SSRP1LA:Os01g0184900	Nfd110:Zm00001d008847	Choi *et al.* (2012)
		SSRP1LB:Os01g0184900		
Pst2	Snl5:At1g59890	Sin3L3:Os01g0109700	Sin3L3:Zm00001d040123	Bowen *et al.* (2010); Choi *et al.* (2012)
	Snl6:At1g10450 ^*c*^			
Rpt3	**Rpt3:At5g58290**	**Rpt3:Os02g0325100**	Zm00001d015886	Kitagawa *et al.* (2014)
Sim3	Nasp:At4g37210	Os07G0122400	Zm00001d007972	Pidoux *et al.* (2003); Dunleavy *et al.* (2007); Le Goff *et al.* (2020)
Spt16	**Spt16:At4g10710**	**Spt16:Os04g0321600**	Spt16:Zm00014a035465	Choi *et al.* (2012)
Spt6	**Gtb1:At1g65440**	**Spt6:Os05g0494900**	Spt6:Zm00001d038570	Choi *et al.* (2012)
	**Spt6:At1g63210**			
*H. sapiens*				
AurkA	**Aur1:At4g32830**	**Os01g0191800**	Zm00001d039498	Kunitoku *et al.* (2003); Slattery *et al.* (2008)
	**Aur2:At2g25880**		Zm00001d008815	
AurkB	Aur3: At2g45490	Aur3:Os03g0765000	Zm00001d034166	Zeitlin *et al.* (2001); Kunitoku *et al.* (2003); Demidov *et al.* (2019)
Bmi-1	Drip1:At1g06770	Drip2:Os12g0600200	Drip2:Zm00001d033322	Obuse *et al.* (2004); Sanchez-Pulido *et al.* (2008)
	Drip2:At2g30580		Zm00001d041405	
			Zm00001d030985	
CenpC	CenpC:At1g15660	CenpCA:Os01g0617700	CenpC:Zm00001d044220	Shibata and Murata (2004); Foltz *et al.* (2006)
CenpU	Bin4:At5g24630	Bin4:Os02g0147700	Bin4:Zm00014a003282	Foltz *et al.* (2006); Kang *et al.* (2011)
Cops8	**Cop9:At4g14110**	**Cop9:Os04g0428900**	Zm00001d003685	Niikura *et al.* (2015)
Cul4-A	Cul4:At5g46210	Cul4:Os03g0786800	Cul4:Zm00001d013116	Niikura *et al.* (2015)
			Zm00001d034361	
Ddb1	**Ddb1a:At4g05420**	Ddb1a:Os05g0592400	Ddb1a:Zm00001d039165	Obuse *et al.* (2004)
	**Ddb1b:At4g21100**			
Ssrp1	**Ssrp1:At3g28730**	Ssrp1LA:Os01g0184900	Nfd110:Zm00001d008847	Foltz *et al.* (2006); Okada *et al.* (2009)
*S. cerevisiae*				
Cdc53	**Cul1:At4g02570**	**Cul1:Os01g0369200**	Cul1:Zm00001d010858	Cheng *et al.* (2016)
Doa1	**At3g18860**	Os07g0123700	Zm00001d018724	Au *et al.* (2013); Cheng *et al.* (2016)
Fun30	Chr19:At2g02090	Os04g0566100	Chr19:Zm00001d002656	Durand-Dubief *et al.* (2012); Narlikar *et al.* (2013)
Gcn5	Gcn5:At3g54610	Gcn5:Os10g0415900	Hag101:Zm00001d014175	Pandey *et al.* (2002); Vernarecci *et al.* (2008)
Hir1	Hira:At3g44530	Os09g0567700	Hira:Zm00001d019789	Sharp *et al.* (2002); Duc *et al.* (2015)
Mcm21	CenpO:At5g10710	Os04g0284100	CenpO:Zm00001d032978	Ranjitkar *et al.* (2010); Samel *et al.* (2012)
Mif2	CenpC:At1g15660	CenpCA:Os01g0617700	CenpC:Zm00001d044220	Pinsky *et al.* (2003); Shibata and Murata (2004); Collins *et al.* (2005); Ranjitkar *et al.* (2010)
Mtw1	Mis12:At5g35520	Mis12:Os02g0620100	Mis12:Zm00001d001797	Pinsky *et al.* (2003); Collins *et al.* (2005); Sato *et al.* (2005); Samel *et al.* (2012)
Ndc80	Ndc80:At3g54630	Os08g0468400	Zm00001d032029	Collins *et al.* (2005); Boeckmann *et al.* (2013); Shin *et al.* (2018)
Pat1	Pat1:At4g14990	Pat1:Os01g0769000	Pat1:Zm00001d038671	Kuromori and Yamamoto (2000); Mishra *et al.* (2015)
	Pat1:At1g79090	Pat1:Os02g0517300	Pat1:Zm00001d043329	
	Pat1:At3g22270			
Psh1	Orth1:At5g39550	Orthus2:Os05g0102600	Zm00001d011108	Ranjitkar *et al.* (2010); Deyter *et al.* (2017); Samel *et al.* (2017
	Orth2:At1g57820 Orth5: At1g66050		Zm00001d035764	Kim *et al.* (2014)
Sgo1	Sgo1:At3g10440	Sgo1:Os02g0799100	Sgo1:Zm00001d019148	Zamariola *et al.* (2013); Buehl *et al.* (2018); Mishra *et al.* (2018)
	Sgo2:At5g04320			
Siz1	Siz1:At5g60410	Os05g0125000	Siz1:Zm00001d010974	Catala *et al.* (2007); Ohkuni *et al.* (2016)
Siz2	Siz1:At5g60410			Catala *et al.* (2007); Ohkuni *et al.* (2016)
Spt16	**Spt16:At4g10710**	Spt16:Os04g0321600	Spt16:Zm00014a035465	Ranjitkar *et al.* (2010)
Sth1	**Chr12:At3g06010**	Os05g0144300	Zm00001d006798	Hsu *et al.* (2003); Ranjitkar *et al.* (2010)
	**Chr23:At5g19310**			
Ubp8	Ubp22:At5g10790	**Upb22:Os04g0647300**		Canzonetta *et al.* (2015)
Ubr2	Prt6:At5g02310	Prt6:Os01g0148000	Zm00001d039860	Samel *et al.* (2017)
		Prt6:Os01g0148050		

^*a*^ No markup, genes identified via reciprocal Blasts from human or yeast to Arabidopsis/rice/maize (no references found).

^*b*^ Bold, genes identified through the NCBI database Homologene.

^*c*^Underlined: genes identified via reciprocal Blasts from human or yeast to Arabidopsis/rice/maize and supported by previous reports (the relevant references are underlined).

Plant orthologs of known interaction partners of CENH3^CENPA/CNP1/CSE^ are considered here as ‘putative conserved interaction partners of CENH3’. In order to find protein homologs in plants, reciprocal protein blasts of human and yeast to plant sequences were performed. The selected candidates were used to perform a literature survey. For the CENH3-interacting proteins HJURP, CENPI, CENPT, CENPM, and CENPP, sequence homology searches did not result in the identification of putative orthologs, indicating poor sequence conservation across species or that plants do not harbor a counterpart. The previous reports suggesting rapid evolution of centromere-associated/kinetochore-related proteins corroborate an apparent lack of sequence conservation (Drinnenberg *et al.*, 2016).

## Candidate CENH3-interacting proteins with functions related to growth and development

The candidate plant orthologs and functional homologs listed in Table 1 have been assigned functions related to different aspects of plant development. Arabidopsis MIS12 (Sato *et al.*, 2005), MSI1 (Hennig *et al.*, 2005), and CUL1 (Shen *et al.*, 2002), for instance, play a critical role in embryo development. Chromosome instability can cause arrests in embryonic development in plants. Therefore, it is also assumed that mutations in CENH3 interaction partners responsible for CENH3 deposition, incorporation, and maintenance cause defects in embryo development. A candidate CENH3-interacting protein required for embryogenesis is MULTICOPY SUPPRESSOR OF IRA 1 (MSI1). MSI1 and MSI1-Like (MSIL) proteins are components of different protein complexes, including the Polycomb Repressive Complex 2 (PRC2) and B-type histone acetyltransferase complexes involved in chromatin remodeling, and pRB (retinoblastoma tumor suppressor protein) that controls the cell cycle and developmental processes (Hennig *et al.*, 2005). MSI1 functions in seed development through interaction with retinoblastoma protein and the CULLIN4–DDB complex, controlling parental gene imprinting, and is a member of the MEDEA (MEA)/FERTILIZATION-INDEPENDENT ENDOSPERM (FIE)/FERTILIZATION- INDEPENDENT SEED2 (FIS) polycomb group complex (Köhler *et al*., 2003; Jullien *et al.*, 2008; Dumbliauskas *et al.*, 2011). MSI1 (Hennig *et al.*, 2005) and CULLIN1 (CUL1) (Shen *et al.*, 2002) play a role in post-embryonic development, and null mutants are embryo lethal, in agreement with a critical role in cell division and development. The plant MSIL protein family (five in Arabidopsis, AtMSI1–AtMSI5, and three in rice, OsRBAP1–OsRBAP3) is larger and more diverse than in fungi, insects, and vertebrates (Yang *et al.*, 2013). While the function of AtMSI2 and AtMSI3 are unknown, AtMSI4/FVE regulate flowering time by repressing FLC expression through a histone deacetylation mechanism (Ausin *et al*., 2004) and play a role in cold stress (Kim *et al.*, 2004). In addition to MSI1L proteins and CUL1, centromere-localized plant MIS12 was shown to be essential for embryogenesis (Sato *et al.*, 2005). The role of these candidate CENH3-interacting proteins in early stages of development suggests a critical role in mitosis, which is in line with the embryo-lethal phenotype of CENH3 knock-out plants (Ravi and Chan 2010) and the root developmental defects reported in plants expressing recombinant CENH3 (Wijnker *et al.*, 2014).

## Candidate CENH3-interacting proteins with functions related to histone chaperones

Nucleosome assembly is mediated by conserved histone chaperones, classified into families based on the founding member genes NAP, CAF1, SPT6, SSRP1, ASF1, HIRA, NASP, and FACT (Tripathi *et al.*, 2015). For several members of these protein families, interaction with CENH3^CENPA/CNP1/CSE4^ has been demonstrated in human and yeast (Table 1). Evidence in plants is largely missing, and only indirect indications for a role in CENH3 chaperone function are available. For instance, the interaction of NASP with both CENH3 and H3.1/H3.3 has been demonstrated (Le Goff *et al.*, 2020). HFDs of H3s and CENH3 show 50–60% sequence similarity within the same species (Talbert and Henikoff, 2010). Considering that HFD plays a role in chromatin targeting, the chaperoning function of NAP, CAF1, ASF1, and HIRA might also be conserved in CENH3 targeting in plants.

In the context of genome elimination, HIRA is a promising candidate for engineering. HIRA activity is specifically impaired in the *Drosophila* mutant *sésame* (*ssm*), causing a unique maternal zygote effect in preventing the formation of the DNA replication-competent male pronucleus, which results in the development of haploid embryos carrying only maternal chromosomes (Loppin *et al.*, 2005). In vertebrates, HIRA is critically involved in nucleosome assembly of the H3.3 histone variant independent of DNA synthesis (Tagami *et al.*, 2004). The replacement of sperm chromosomal proteins by maternally provided histones is impaired in *ssm*, in agreement with the histone chaperone protein function of HIRA (Loppin *et al.*, 2005). While the *A. thaliana* HIRA protein interacts with H3.3, a knock-out mutant displays only a mild growth phenotype and does not affect sexual reproduction and embryogenesis, suggesting that plant HIRA has diversified to function during sporophytic development (Nie *et al.*, 2014). A weak sexual reproduction phenotype was, however, reported for a *hira* transposon mutant (same as in the study by Nie *et al.*, 2014) and, combined with the *fas1-4* mutation, the double mutant did not produce viable pollen (Duc *et al.*, 2015). ASYMMETRIC LEAVES 2 (AS2) has been shown to repress the meristem development gene KNOTTED1-like homeobox (KNOX) during organogenesis through the interaction with histone chaperone HIRA (Guo *et al.*, 2008). In view of the role of HIRA in controlling the expression of KNOX genes through binding with the transcription factors AS1 and AS2 (Guo *et al.*, 2008), it seems that HIRA has a complex function in cell growth and development. It is currently not clear how this is linked with H3.3 nucleosome assembly.

## Candidate CENH3-interacting proteins with functions related to DNA damage

A possible role for CENH3 in DNA damage response in mammals has been proposed based on the observation that CENH3^CENPA^ and other centromeric proteins are recruited to double-strand breaks (Zeitlin *et al.*, 2009). CENH3 also accumulates at neocentromeres that are formed at DNA breakpoints (Hasson *et al.*, 2011) and in conditions causing genomic rearrangements such as in wide species crosses (Cuacos *et al.*, 2015), suggesting that CENH3 functioning is somehow associated with DNA damage. In CENH3-based haploid induction in plants, the selective loss of chromosomes is accompanied by major chromosome rearrangements relying on the DNA repair enzyme DNA ligase 4 (Tan *et al.*, 2015). Some chromosome fragments are transmitted to the next generation and are reintegrated into the genome by a DNA damage repair mechanism (Comai and Tan, 2019). Whether CENH3 is linked to the unknown mechanism behind the activation of the DNA damage response pathway following the chromosome elimination remains to be tested.

Genome instability upon UV-induced double-strand breaks triggers the highly conserved DAMAGE DNA BINDING (DDB1) proteins DDB1A and DDB1B to form a complex with CULLIN4 (CUL4) (Molinier *et al.*, 2008; Ganpudi and Schroeder, 2013). The loss of DDB1B results in embryo lethality, indicating that these regulators are also important for basic functions in the absence of stress (Bernhardt *et al.*, 2010). DDB1A physically interacts with MSI1, thereby regulating the PRC2 complex that controls imprinting and endosperm development (Dumbliauskas *et al.*, 2011). In plants, a link between CENH3 in DNA damage response pathways has so far not been reported. The fact that CENH3-interacting animal and yeast proteins involved in DNA damage response are conserved in plants calls for investigating a presumptive role for CENH3 in the CUL4, DDB1A, or DDB1B and MSI1 controlled DNA damage response.

## Post-translational modifications of CENH3

**Fig. 1.**
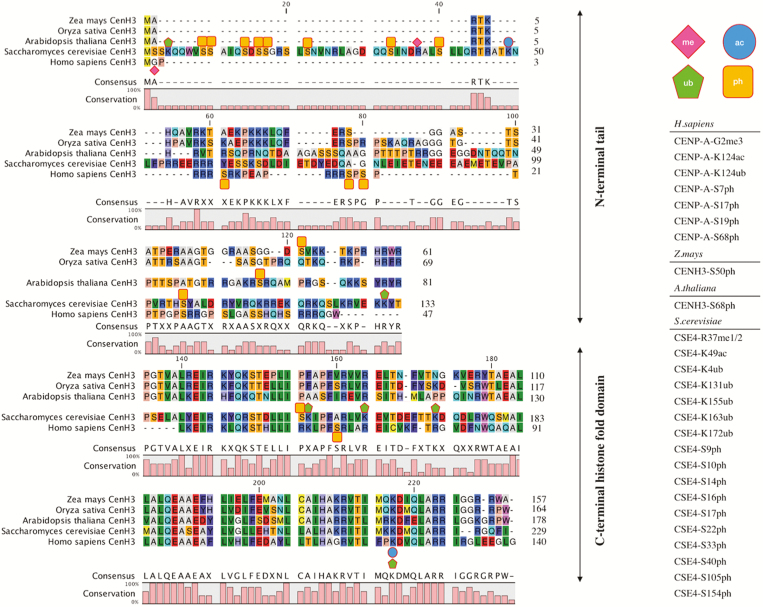
Model organism CENH3 amino acid sequence and reported PTMs. The *A. thaliana*, *O. sativa*, *Z. mays*, *S. cerevisiae*, and *H. sapiens* CENH3 sequences are shown with the existing identified post-translational modifications (me, methylation; ac, acetylation; ub, ubiquitination; ph, phosphorylation) on *S. cerevisiae*, *H. sapiens*, *Z. mays* (Zm), and *A. thaliana* (At) CENH3. PTMs listed here are reported in Zeitlin *et al.* (2001; CENPA-S7ph); Zhang *et al.* (2005; ZmCENH3-S50ph); Hewawasam *et al.* (2010; CSE4-K4ub, CSE4-K131ub, CSE4-K155ub, CSE4-K163ub, and CSE4-K172ub); Samel *et al.* (2012; CSE4-R37me1/2); Bui *et al.* (2012; CENPA-K124ac); Bailey *et al.* (2013; CENPA-G2me3, CENPA-S17ph, and CENPA-S19ph); Boeckmann *et al.* (2013; CSE4-K49ac, CSE4-S22ph, CSE4-K33ph, CSE4-S40ph, and CSE4-S105ph); Niikura *et al.* (2015; CENPA-K124ub); Yu *et al.* (2015; CENPA-S68ph); Mishra *et al.* (2019; CSE4-S9ph, CSE4-S10ph, CSE4-S14ph, CSE4-S16ph, CSE4-S17, and CSE4-S154ph); and Demidov *et al.* (2019; AtCENH3-S68ph).

Chromatin displays local DNA and histone modification patterns shaping the structural organization and stability of protein–nucleosome–DNA interactions. The histones are subjected to a variety of post-translational modifications (PTMs) including addition of methyl, acetyl, ubiquitin, phosphoryl, and ADP-ribosyl groups that influence their interaction with axillary factors, many of which are regulating gene expression (Rothbart and Strahl, 2014). CENH3 PTM serves other functions such as the maintenance of centromeric nucleosomes (Niikura *et al.*, 2015). An alignment of CENH3 from *Saccharomyces cerevisiae*, *Homo sapiens*, *A. thaliana*, *Z. mays*, and *O. sativa* reveals multiple candidate PTM sites in plants, many of which have been reported to undergo ubiquitination, acetylation, phosphorylation, and methylation (Fig. 1).

The HFD of CENH3^CENPA^ contains an acetylated or ubiquitinated lysine residue (CENPA-K124) that is conserved in the five aligned centromeric histone sequences (Bui *et al.*, 2012; Niikura *et al.*, 2015). Ubiquitination at that position in human cells depends on COPS8, a gene conserved in plants (Table 1), and functions in ubiquitin-mediated protein degradation as a component of the COP9 signalosome (Schwechheimer and Isono, 2010). Plant development is orchestrated via components of the COP9 signalosome by controlling proteolysis in adjacent developmental stages (Qin *et al.*, 2020). As an important element of cell division, CENH3 deposition and maintenance at the centromeres can also be regulated as a part of the COP9 signalosome. Such regulation would give plants flexibility to cease or proceed with cell division to fulfill the requirement of different developmental stages.

Ubiquitination of CENH3 plays an important role in the stability of incorporated CENH3^CSE4^ at the centromeres in yeast (Hewawasam *et al.*, 2010; Au *et al.*, 2013) and CENH3^CENPA^ deposition in animal cells (Niikura *et al.*, 2015), albeit that some modifications are dispensable for the long-term function and identity of the centromeres (Fachinetti *et al.*, 2017). The ubiquitination-dependent proteolytic degradation of CENH3^CSE4^ is clearly established in yeast. In *S. cerevisiae*, PSH1 is an E3 ubiquitin ligase controlling the stability and localization of CENH3^CSE4^ by targeting the C-terminus for ubiquitination, and is required for chromosome segregation (Hewawasam *et al.*, 2010). An analogous function of PSH1 is executed by the *A. thaliana* ORTH/VIM proteins that function redundantly as ubiquitin ligases and regulate epigenetic silencing by modulating DNA methylation and histone modification (Woo *et al.*, 2007; Kraft *et al.*, 2008; Kim *et al.*, 2014). VIM1 interacts with CENH3 *in vivo* in *A. thaliana*, and is required for maintenance of centromere DNA methylation and proper interphase centromere organization (Woo *et al.*, 2008).

Several phosphorylation sites have been identified in CENH3^CENPA^ of which S7 is phosphorylated by Aurora kinase, and plays an unexpected role in cytokinesis (Zeitlin *et al.*, 2001). Cell cycle-dependent phosphorylation of CENH3^CENPA^ is mediated by cyclin E1/CDK2 at S18 (Takada *et al.*, 2017). In maize CENH3 is also phosphorylated in a cell cycle-dependent fashion at position S50 (Zhang *et al.*, 2005). A recent study shows that Aurora3 phosphorylates Arabidopsis CENH3 at S65 (Demidov *et al.*, 2019). Phosphorylation of S65 of CENH3 occurs in different developmental stages of Arabidopsis yet this PTM is mainly linked with floral meristem development. Further studies are required to determine what function phosphorylation of CENH3 plays in cell division.

Poly(ADP-ribose) polymerases (PARPs) are responsible for ADP-ribosylation of CENH3^CENPA^ (Saxena *et al.*, 2002) and are conserved in plants (*A. thaliana* PARP1:At2g31320, *O. sativa* PARP1:Os07g0413700, and *Z. mays* PARP1:Zm00001d005168). PARP was shown to bind the 180 bp centromeric repeat sequence from Arabidopsis, suggesting that it may be independently targeted to the centromeres (Babiychuk *et al.*, 2001). PARP plays a role in the DNA damage response and hence its association with CENH3 should be seen in the context of stress and UV DNA damage.

## Conclusion

In view of the role of recombinant CENH3 in chromosome elimination and the development of methods to generate haploids for plant breeding, we point out the importance of identifying CENH3 interaction partners. A list of putative plant orthologs and functional homologs of animal and yeast CENH3-binding proteins is presented that serves as a starting point for further research. CENH3-interacting proteins are involved in a variety of biological pathways and many are putatively involved in chemically modifying CENH3. The conservation of these genes suggests that plant CENH3 undergoes similar PTMs. Whether any of these modifications are involved in chromosome elimination remains to be discovered.
